# A Guide to Implementing Immune Checkpoint Inhibitors within a Cancer Program: Experience from a Large Canadian Community Centre

**DOI:** 10.3390/curroncol29020074

**Published:** 2022-02-04

**Authors:** Parneet K. Cheema, Marco A. J. Iafolla, Massey Nematollahi, FeRevelyn Berco, Deepanjali Kaushik, Priscilla Matthews, William R. Raskin, Kirstin A. Perdrizet, Shaan Dudani, Juhi Husain, Margaret Balcewicz, Philip G. Kuruvilla, Stephen M. Reingold, Henry J. Conter

**Affiliations:** 1 Medical Oncology/Hematology, William Osler Health System, Brampton, ON L6R 3J7, Canada; marco.iafolla@williamoslerhs.ca (M.A.J.I.); massey.cns@gmail.com (M.N.); ferevelyn.berco@williamoslerhs.ca (F.B.); deepanjali.kaushik@williamoslerhs.ca (D.K.); priscilla.matthews@williamoslerhs.ca (P.M.); william.raskin@williamoslerhs.ca (W.R.R.); kirstin.perdrizet@williamoslerhs.ca (K.A.P.); shaan.dudani@williamoslerhs.ca (S.D.); juhi.husain@williamoslerhs.ca (J.H.); margaret.balcewicz@williamoslerhs.ca (M.B.); philip.kuruvilla@williamoslerhs.ca (P.G.K.); stephen.reingold@williamoslerhs.ca (S.M.R.); henry.conter@williamoslerhs.ca (H.J.C.); 2 Department of Medicine, University of Toronto, Toronto, ON M5S 1A8, Canada; 3 Department of Oncology, Western University, London, ON N6A 5W9, Canada

**Keywords:** immune checkpoint inhibitors, immune-related adverse events, cancer immunotherapy, patient education, multidisciplinary teams

## Abstract

The increased use of immune checkpoint inhibitors across cancer programs has created the need for standardized patient assessment, education, monitoring, and management of immune-related adverse events (irAEs). At William Osler Health System in Brampton, Ontario, a practical step-wise approach detailing the implementation of cancer immunotherapy in routine practice was developed. The approach focuses on four key steps: (1) identification of patient educators; (2) development of patient education materials; (3) development of patient monitoring tools; (4) involvement and education of multidisciplinary teams. Here, we provide an in-depth description of what was included in each step and how we integrated the different elements of the program. For each step, resources, tools, and materials that may be useful for patients, healthcare providers, and multidisciplinary teams were developed or modified based on existing materials. At our centre, the program led to improved patient comprehension of irAEs, the ability to act on symptoms (patient self-efficacy), and low rates of emergency room visits at first presentation for irAEs. We recognize that centres may need to tailor the approaches to their institutional policies and encourage centres to adapt and modify the forms and tools according to their needs and requirements.

## 1. Introduction

Immune checkpoints such as cytotoxic T-lymphocyte antigen 4 (CTLA-4), programmed cell death 1 (PD-1), and programmed cell death ligand 1 (PDL-1) are negative regulators of immune activation, allowing cancer cells to evade immune surveillance, thereby enabling unchecked tumour growth [1]. Immune checkpoint inhibitors (ICIs) negate this key mechanism of cancer progression, restoring the immune system’s ability to eradicate cancer cells; however, releasing the effects of the immune system can result in a unique spectrum of toxicities known as immune-related adverse events (irAEs) [2,3]. Dermatological, gastrointestinal, and endocrine irAEs are the most common types of manifestations. Unlike the predictable nadir or cyclic profile of AEs associated with conventional chemotherapy, irAEs are unpredictable, heterogeneous, and in some instances irreversible or life-threatening. The frequency of irAEs also varies depending on the type of ICIs and their mechanism of action. Furthermore, irAE onset can occur during treatment or following treatment completion, and multiple organs may be simultaneously or separately affected at different time points.

The overall incidence of all grades of irAEs may be as high as 75% in patients treated with CTLA-4 inhibitors [4] and up to 30% in patients treated with PD-1/PD-L1 inhibitors [5,6,7]. CTLA-4 inhibitor-related irAEs are generally dose-dependent and increase with the higher CTLA-4 inhibitor dose [8]. Although less frequent, grade 3–4 irAEs occur in up to 15–20% of patients treated with single-agent PD-1/PD-L1 inhibitor. Furthermore, irAEs associated with PD1/PD-L1 inhibitors do not appear to be dose-dependent [5,9,10]. Combined treatment with CTLA-4 and PD-1 or PD-L1 inhibitors led to irAE rates of 95% (59% grade 3 or 4) [11].

Although the majority of irAEs are mild or moderate in severity, fatal outcomes of irAEs were reported in 0.36% and 1.23% of patients treated with PD-1 and PD-1/PD-L1 plus CTLA-4 inhibitors, respectively [12]. While pneumonitis was the major cause (35%) of all fatal irAEs in patients on PD-1 inhibitors, 70% of fatal irAEs in patients treated with the combination therapy (PD-1/PD-L1 plus CTLA-4 inhibitors) were due to colitis. Fatal adverse effects typically occurred early after therapy initiation.

The management of irAEs is based on well-established clinical practice guidelines, such as the National Comprehensive Cancer Network (NCCN) [13], the American Society of Clinical Oncology (ASCO) [14] the European Society for Medical Oncology (ESMO) [15], the Society for Immunotherapy of Cancer (SITC) [16], and Cancer Care Ontario [17]. These guidelines strongly recommend multidisciplinary collaboration.

The basic principles of irAE management include education of patients and caregivers about immunotherapy and mechanisms of action, as well as the clinical signs and symptoms of possible irAEs throughout treatment [18]. Detailed knowledge of potential side effects is important for the early recognition of irAEs, as it can lead to timely treatment that may reverse organ dysfunction, and in some cases prevent fatal outcomes. Education is not only important for patients and caregivers—all healthcare professionals involved in the care of patients receiving immunotherapy should be aware of the rare or subtle symptoms of irAEs.

Although clinical practice guidelines provide high-level recommendations for the management of irAEs, institutional implementation presents unique challenges due to a lack of practical protocols and algorithms that have been successfully followed. Providing examples of feasible algorithms from different institutions and background information about the development processes that took place can be beneficial for other cancer programs to adapt similar proven models. Moreover, individual institutions have unique configurations with regards to multidisciplinary collaboration that is largely driven by available resources, allocated funding, and monitoring capacities. General principles and recommendations outlined by regional, national, and international guidelines should be further adapted to meet institutional standards and capabilities; however, busy clinical practices often pose significant burdens on treating clinicians and prevent the development of appropriate irAE-related algorithms tailored to their institution.

Here we describe the processes that led to the development of the Osler Cancer Immunotherapy Program (OCIP), which focused on the standardization of patient education and monitoring of patients prescribed ICIs. The program was implemented at the William Osler Health System (Osler), a hospital network in Ontario, Canada, which serves 1.3 million residents. Although services are provided through three facilities, the centre strives to provide a single standard of care. Since inception, over 400 patients prescribed ICIs have benefited from our assessment, education, and monitoring program.

The approach was initially evaluated in a prospective study (May 2018–December 2019) with 80 patients [19]. The Cancer Behaviour Inventory—Brief Version (CBI-B) [20,21] was used to evaluate patient comprehension of irAEs and self-efficacy (patient ability to act on symptoms). The median follow-up at 4.1 months demonstrated significant improvements in average baseline CBI-B scores (*p* < 0.001) that were maintained over time. In total, 62% of irAEs were detected by self-reporting and 27% during proactive callbacks by the immunotherapy nurse. Only 3 patients (4%) had an irAE at first presentation detected during an emergency room visit and there were low rates of emergency room visits for irAEs.

By describing the processes and providing the algorithms and protocols implemented at Osler, our goal is to inform and assist other institutions in developing similar pathways. All materials are openly available as Supplementary Materials online at https://doi.org/10.6084/m9.figshare.c.5735318. Files in the repository are continuously updated, and additional files are added (e.g., translated materials) as they are developed and implemented in the OCIP. We encourage and invite others to adapt the materials developed at our centre according to their needs and standards—including our “A Guide to Implementing Cancer Immunotherapy at Your Centre” (File S01).

When possible, we also leverage materials from the Community Immunooncology Support Kit (CIOSK), which are materials generated via the collaboration of health practitioners across Canada.

## 2. Implemented Pathways and Ongoing Initiatives

The four principles applied by the OCIP include: (1) identification of patient educator(s); (2) development of patient education materials; (3) development of patient monitoring tools; (4) involvement and education of multidisciplinary teams.

### 2.1. Identification of Patient Educator(s)

Patient education is a critical component of the management of irAEs. Patients must be educated about the therapies they are receiving (generic and brand names as well as the mechanisms of action and potential toxicities) and be able to recognize toxicity-related symptoms, especially those that may require urgent medical attention. They must also know how to, when to, and to whom to report symptoms.

Thus, the first step in providing consistent and high-quality care to patients starting on ICIs is to identify patient educators. At our centre, the role was assumed by an immunotherapy nurse. The role encompasses education, and also includes an extensive baseline patient assessment, proactive callbacks, and timely communication with the multidisciplinary team as required. Additionally, we identified an oncologist physician lead with expertise in managing irAEs to assist colleagues with difficult cases.

### 2.2. Development of Patient Education Materials

In our next step, we reviewed existing patient education and counselling materials and identified gaps for which materials needed to be developed. Patient education materials included printed materials and videos designed to assist with educating patients and their families on the basics of immunotherapy, emphasizing signs and symptoms of irAEs.

The patient education and assessment process at Osler includes a one-on-one session between the patient and the immunotherapy nurse. During the session, the nurse goes through our novel comprehensive baseline assessment (New Patient Immunotherapy Baseline Assessment and Teaching Checklist; Table 1; File S02).

The baseline assessment is used to document baseline symptoms to be able to establish a change in the patient’s clinical status when working up for an irAE, as well as to determine risks for irAEs. Patients deemed at high risk of developing an irAE receive proactive weekly callbacks. High-risk patients are those with a risk greater or equal to 20% of developing a grade 3 or 4 irAE (this includes patients prescribed an CTLA-4 inhibitor, with autoimmune condition, or who have had rechallenge after a previous irAE). Baseline symptoms are simultaneously documented using the CIOSK New Patient Baseline Symptom Tracker Sheet (File S03), which was adapted from CIOSK patient materials (Files S03–S08).

**Figure 1:**
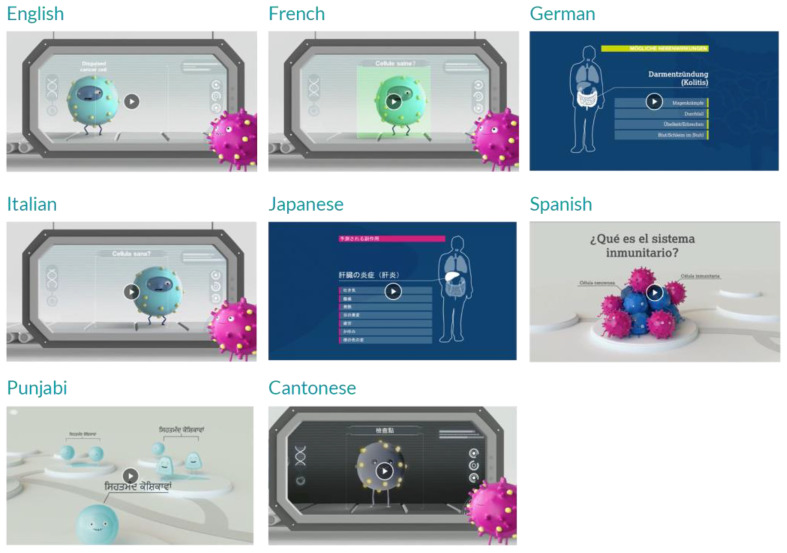
Multilingual patient education videos.

**Figure 2:**
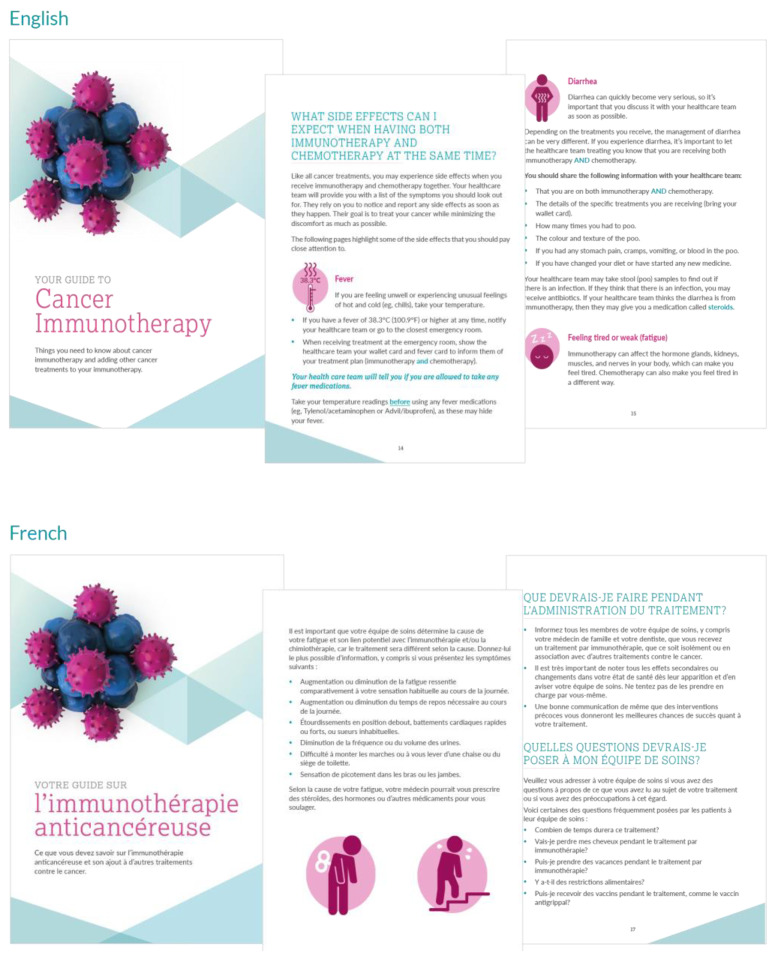
Patient immunotherapy education booklet.

Patients are then shown a 6 minute teaching video in their language of choice. This video was created by our team in collaboration with healthcare workers across Canada and patient focus groups, acknowledging the cultural diversity in our population by translating the video into 8 different languages (English, French, German, Italian, Japanese, Spanish, Punjabi, Cantonese; Figure 1; Videos S01–S08). Patients are sent home with a paired cancer immunotherapy booklet called “Your Guide to Cancer Immunotherapy” (Figure 2; File S09), which is intended to provide further reinforcement of concepts learned from the immunotherapy video and to fill gaps in education for patients receiving ICIs with other anti-cancer therapies (e.g., chemotherapy).

In addition, patients receive materials adapted from the CIOSK program, a wallet card (File S04), and a symptom diary (File S05). The patient’s wallet card is intended to provide information to healthcare providers not involved with the patient’s cancer treatment, such as their primary care provider or an emergency physician. Similarly, a letter is provided to patients for their general practitioner—it communicates that the patient will be starting immunotherapy and further educates local family physicians on immunotherapy and irAEs (File S06). Drug-specific information sheets are also provided. Furthermore, in each of the patient exam rooms, there is a patient-facing poster of irAEs (File S07).

### 2.3. Development of Patient Monitoring Tools

Baseline and ongoing patient monitoring tools were developed based on standard assessments suggested by the SITC working group [16] and in collaboration with our multidisciplinary team. In addition to baseline assessments, specific biochemical monitoring recommendations based on the type of immunotherapy (i.e., anti-CTLA4 monotherapy or in combination with anti-PD-1/PD-L1 vs. anti-PD-1/PD-L1 monotherapy) were developed (Table 2).

Prior to ICI treatment initiation, all patients are assessed for underlying conditions that may increase their risk of irAEs and need for high-dose immunosuppressants. At the recommendation of our infectious disease specialist and respirologists, patients at high risk of developing an irAE are screened for tuberculosis using the Mantoux tuberculin skin test in the event there is urgent need for immunosuppressants.

As per ongoing monitoring, all high-risk patients have proactive weekly calls with the immunotherapy nurse. The standardized patient monitoring questionnaire (File S08) is designed to assess endocrine, pulmonary, hepatic, gastrointestinal, and skin-related symptoms associated with irAEs, as well as patients’ overall wellbeing.

### 2.4. Involvement and Education of Multidisciplinary Teams

As per published guidelines, the involvement of a multidisciplinary team is required for the management of irAEs. Table 3 outlines the steps in the development and implementation of the multidisciplinary approach for the management of irAEs at Osler.

The need for multidisciplinary collaboration at the institution level was addressed at a working group meeting in 2018. Prior to the meeting, we identified unmet needs and areas where additional efforts and improvement were needed. To that end, a brief survey (via SurveyMonkey^®^) was sent to 11 different specialists involved in the management of irAEs—a medical oncologist, oncology nurse practitioner, respirologist, endocrinologist, gastroenterologist, rheumatologist, ophthalmologist, nephrologist, neurologist, cardiologist, and infectious disease specialist. The survey revealed that the specialists had a good overall knowledge of irAEs; however, some areas of uncertainty indicated a need to further streamline the processes and multidisciplinary collaborations. The objectives of the working group meeting were to: (1) assess current management and challenges in treating irAEs; (2) develop standardized templates for diagnostic workup, recognition, and referral for consultation for patients with suspected irAEs; (3) create treatment algorithms for managing irAEs. During the meeting, each subspecialist provided input on clinical data and laboratory investigations required for rapid assessment and diagnosis of organ-specific irAEs.

**Figure 3:**
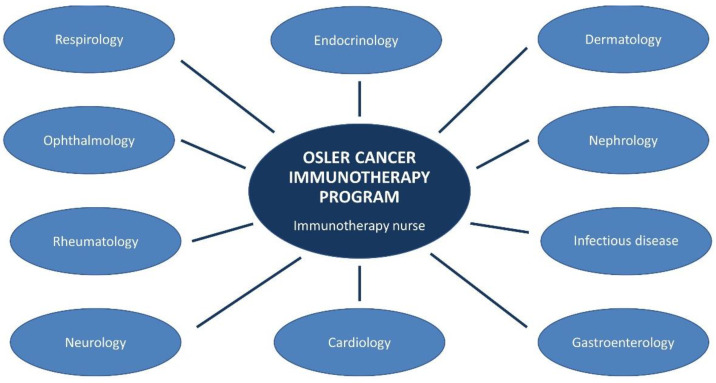
Multidisciplinary network for rapid assessment and treatment of irAEs at Osler.

Implementing a multidisciplinary approach and pathways at the institutional level is key for the early recognition and appropriate management of irAEs, as well as for outpatient management. The regional multidisciplinary network for rapid outpatient assessment and treatment of irAEs at Osler consists of 10 non-oncology subspecialties (Figure 3). They provided input on how to screen and monitor patients for irAEs for their respective organs. In addition, they are local champions who educate on their own subspecialty of irAEs.

The multidisciplinary collaboration led to the development of irAE-specific referral forms with clinical information required for subspecialty consultations, as well as ways to expedite the outpatient referral process and necessary consultations. In the majority of cases, discussions regarding the stabilization of a patient needing urgent care occur immediately. Patients needing further assessment are seen by the specialist within a target timeframe of 1–2 weeks, and any interventions such as endoscopy and bronchoscopy are performed during that timeline.

Further to the program, we created standardized step-by-step guidelines for our institution on the management of select irAEs, with accepted published guidelines incorporated.

## 3. Discussion and Conclusions

It is well accepted and understood that the achievement of optimal outcomes in patients undergoing treatment with ICIs requires immunotherapy-specific education and monitoring of patients, with a multidisciplinary approach in order to recognize and manage irAEs in a timely and effective manner. To that end, various regional, national, and international guidelines and working groups provide a high level of recommendations for innovative institutional solutions for the management of irAEs [13,14,15,16,17,22]; however, the recommendations lack practical “how-to” approaches and tips. This manuscript provides detailed information and insights on what was done at Osler and how it was done, as we endeavoured to address some of the gaps and challenges at our centre. Furthermore, we demonstrate how to follow the guideline recommendations in a practical manner.

For example, the guidelines recommend that the first steps in the management of irAEs are patient-related initiatives. These include patient education and identification of patients who will receive ICI-based therapy. We addressed these recommendations by providing patient education in different languages and using different means of communication—from verbal and written instruction to educational videos and individualized identification cards.

Another approach recommended by the guidelines is engaging designated irAE response teams consisting of medical oncologists, nurses, and relevant medical specialists outside the oncology discipline. Such multidisciplinary teams should provide guidance and consultation for any affected patient. Our example demonstrates that although challenging, such multidisciplinary rapid out-patient pathways for the work up and management of irAEs can be successfully implemented. We expect that with the increased use of ICIs, the specialized management of irAEs at our centre will further improve and become a practical skill within each subspecialty involved.

The development of pathways for the management of irAEs at our centre is an ongoing dynamic process that will evolve as new data and treatments are available. With key principles that include patient education, initial assessment, and ongoing monitoring, as well as referral pathways and multidisciplinary collaborations in place, we are confident that we are well prepared to identify and overcome new challenges that are likely to occur as cancer treatment and the use of immune-based therapies continue to advance.

We plan to continue publishing and sharing our experience with both oncology and non-oncology providers, regionally and beyond, with the aim of improving clinician awareness and expertise and assisting in building institutional safety standards and processes that will ultimately lead to improvements in patient outcomes.

We encourage other centres to utilize the materials we developed or the CIOSK materials to adapt them according to their needs. At our centre, the program led to improved patient comprehension of irAEs, the ability to act on symptoms (patient self-efficacy), and low rates of emergency room visits for irAEs. We hope that other institutions will find the materials beneficial and have similar experiences in regard to patient outcomes.

## Figures and Tables

**Table 1:** New Patient Immunotherapy Baseline Assessment and Teaching Checklist.

(A) Baseline Assessment				
1. Vital signs	Blood pressure Peripheral capillary oxygen saturation Weight and height			
2. Bowel movement habits	Number of bowel movements per day Consistency of bowel movements			
3. Biochemical monitoring	Is all blood work completed? If not, has missing lab work been ordered? Identify patients with chronic infections (Hep B, Hep C, HIV) and refer them to immunotherapy-program-affiliated infectious disease specialist.			
4. History of autoimmune disease	Endocrine: ☐ Addison disease ☐ Graves disease ☐ Hashimoto thyroiditis ☐ Hypophysitis ☐ Type 1 diabetes ☐ Other:____________	Rheumatologic: ☐ Rheumatoid arthritis ☐ Scleroderma ☐ Sjögren’s syndrome ☐ Systemic lupus erythematosus ☐ Other non-RA inflammatory arthritis ☐ Other:____________	Neurologic/neuromuscular: ☐ Guillain-Barre syndrome ☐ Multiple sclerosis ☐ Myasthenia gravis ☐ Myopathy ☐ Myositis ☐ Peripheral neuropathy ☐ Other:____________	Lung: ☐ Interstitial lung disease ☐ Interstitial pneumonitis ☐ Other:____________
	Gastrointestinal: ☐ Celiac disease ☐ Crohn’s disease ☐ Hepatitis ☐ Ulcerative colitis ☐ Other:____________	Dermatologic: ☐ Bullous pemphigus ☐ Eczema ☐ Paraneoplastic rash ☐ Psoriasis ☐ Other:____________	Organ transplant: ☐ History of prior organ transplant and immunosuppressant therapy ☐ Other:____________________________	
	For patients with pre-existing autoimmune conditions, ensure documentation from treating subspecialist aware of treatment plan, or facilitate referral to appropriate subspecialist through immunotherapy program network.			
	For patients with pre-existing autoimmune conditions on immunosuppressants, ensure documentation from oncologist and/or treating specialist of plan regarding immunosuppressants while receiving immunotherapy.			
5. Completed patient baseline symptoms (symptom tracker sheet)				
6. (A) Identify high-risk patients: If any of the first three boxes are checked, patient is categorized as high risk. Proceed to 6b. If ‘None of the above’ is selected, skip 6b. If ‘Other’ is selected, skip 6b, but determine whether patient should receive callbacks. ☐ Patients receiving anti-CTLA4 monotherapy or in combination ☐ Patients with pre-existing autoimmune condition that increases risk of requiring immunosuppressants ☐ Re-challenging immunotherapy in patients that had a previous irAE ☐ None of the above ☐ Other:___________________________				
(B) Baseline Mantoux for High-risk Patients If Mantoux test indicated, identify any contradictions to Mantoux test ☐ Known history of latent or active TB ☐ BCG vaccine ☐ Allergy to components of Mantoux test ☐ None of the above ☐ Not indicated recent Mantoux test Completion date:___________________________ Results:___________________________ Read by:___________________________________ Date:______________________________ If positive Mantoux test, referral to immunotherapy-program-affiliated respirologist or infectious disease specialist.				
(B) Medication History				
☐ Medication reconciliation: Including over-the-counter (OTC), topical steroid, and herbal medications. Inform pharmacist for review of best possible medication history (BPMH). ☐ Recent vaccination(s) (<1 year):_____________________________________________________________				
(C) Teaching				
☐ Review patient adverse events education sheet ☐ Patient watched the patient education video (“What is Immunotherapy?”)—language-specific ☐ Review the cancer immunotherapy patient education booklet (“Your Guide to Cancer Immunotherapy”) ☐ Review immunotherapy-specific drug materials and scheduling with patients ☐ Provide patients immunotherapy patient package, which includes: ☐ Drug-specific patient information sheets ☐ Cancer immunotherapy patient education booklet (“Your Guide to Cancer Immunotherapy”) ☐ Patient education video card (“What is Immunotherapy?”) ☐ Patient adverse event education sheet ☐ Patient symptom diary ☐ Contact numbers (include after hours) ☐ Patient wallet card ☐ Fever card (if concurrent chemotherapy)				
(D) Proactive Follow-up Plan				
☐ Identify proactive follow-up plan ☐ Standard anti-PD-1/PD-L1 monotherapy ☐ High-risk ☐ Weekly proactive call-backs Close blood work monitoring for patients on anti-CTLA4, cycles 2, 3, 4, and 8 weeks post anti-CTLA4 ☐ Inform primary care physician that patient is starting immunotherapy ☐ Fax “Dear Doctor” letter to patient’s primary care physician ☐ Patient provided letter to give to primary care physician				

**Table 2:** Standardized biochemical monitoring.

Baseline	Monitoring Anti-CTLA4 Monotherapy or in Combination with Anti-PD-1/PD-L1 *	Monitoring Monotherapy Anti-PD-1/PD-L1
CBC	CBC	CBC
Chemistry: Creatinine, sodium, potassium, calcium, magnesium, phosphate	Chemistry: Creatinine, sodium, potassium, calcium, magnesium, phosphate	Chemistry: Creatinine, sodium, potassium, calcium, magnesium, phosphate
Liver function: Total bilirubin, ALT	Liver function: Total bilirubin, ALT	Liver function: Total bilirubin, ALT
Endocrine: TSH, T4, morning cortisol, ACTH, prolactin, testosterone (males), FSH/LH (females)	Endocrine: TSH, T4, morning cortisol, ACTH, prolactin, testosterone (males), FSH/LH (females)	Endocrine: TSH, T4 (each cycle to 3 months, then Q1-3 monthly per physician discretion)
Neurologic: CK	Neurologic: CK	-
Urinalysis	Urinalysis	-
Virology: Hepatitis B surface antigen, hepatitis B core antibody, hepatitis C antibody, HIV serology	-	-
Mantoux testing in those deemed high-risk without contraindications to testing.	-	-

* Continue monitoring with the anti-CTLA4 blood work until 8 weeks post last dose of anti-CTLA4, as per CTLA4 pathway, then move to monitoring monotherapy anti-PD-1/PD-L1. ACTH, adrenocorticotropic hormone; ALT, alanine aminotransferase; CBC, complete blood count; CK, creatine kinase; FSH, follicle stimulating hormone; LH, luteinizing hormone; TSH, thyroid-stimulating hormone; T4, thyroxine.

**Table 3:** Implementing a multidisciplinary approach for the management of irAEs withrecommendations based on OCIP experience.

Step	Description
1	Identify relevant medical subspecialists beyond medical oncologists who have an interest, understanding, and willingness to assist in the management of irAEs.
2	Conduct a brief survey to identify potential unmet needs and specific challenges that these specialists may have regarding the management of irAEs.
3	Organize a working group meeting with identified specialists to develop a set of centre- and organ-specific recommendations and pathways.
4	Continue collaboration between the medical oncology team and identified specialists in developing and improving referral pathways for early recognition and management of irAEs.
5	Provide easy access to all developed materials (from patient education to referral forms and algorithms) at institutional level and beyond for the benefit of others.

## Data Availability

The data discussed herein are available within the article and its references. See supplementary materials section above for OCIP materials.

## Supplementary Materials

The following are available online at https://www.mdpi.com/article/10.3390/curroncol29020074/s1. All materials are openly available as supplementary files. Files in the repository will be updated, and additional files will be added (e.g., translated materials) as they are developed and implemented in the OCIP. The following files are available online at https://doi.org/10.6084/m9.figshare.c.5735318: File S01: A Guide to Implementing Immunotherapy at Your Centre. File S02: New Patient Immunotherapy Baseline Assessment and Teaching Checklist. File S03: CIOSK New Patient Baseline Symptom Tracker Sheet. File S04: CIOSK Patient Wallet Card. File S05: CIOSK Patient Symptom Diary. File S06: CIOSK Dear Dr. Letter. File S07: CIOSK Clinic Rooms Poster. File S08: CIOSK Patient Monitoring Questionnaire. Video S01: What is Immunotherapy? (English). Video S02: What is Immunotherapy? (French). Video S03: What is Immunotherapy? (German). Video S04: What is Immunotherapy? (Italian). Video S05: What is Immunotherapy? (Japanese). Video S06: What is Immunotherapy? (Spanish). Video S07: What is Immunotherapy? (Punjabi). Video S08: What is Immunotherapy? (Cantonese). File S09: Your Guide to Cancer Immunotherapy.
