# Supplementary material for: A Guide to Implementing Immune Checkpoint Inhibitors within a Cancer Program: Experience from a Large Canadian Community Centre
Source: Curr Oncol. 2022 Feb 4;29(2):869–80. doi: 10.3390/curroncol29020074 (PMC8870472; doi:10.3390/curroncol29020074)
Supplement: Supplementary file 1 [file curroncol-29-00074-s001.zip › curroncol-1545725-supplementary.pdf]

Supplementary materials

All materials are openly available as supplementary files. Files in the repository will be updated, and additional files will be added (e.g., translated materials) as they are developed and implemented in the OCIP. The following files are available online at https://doi.org/10.6084/m9.figshare.c.5735318: File S01: A Guide to Implementing Immunotherapy at Your Centre. File S02: New Patient Immunotherapy Baseline Assessment and Teaching Checklist. File S03: CIOSK New Patient Baseline Symptom Tracker Sheet. File S04: CIOSK Patient Wallet Card. File S05: CIOSK Patient Symptom Diary. File S06: CIOSK Dear Dr. Letter. File S07: CIOSK Clinic Rooms Poster. File S08: CIOSK Patient Monitoring Questionnaire. Video S01: What is Immunotherapy? (English). Video S02: What is Immunotherapy? (French). Video S03: What is Immunotherapy? (German). Video S04: What is Immunotherapy? (Italian). Video S05: What is Immunotherapy? (Japanese). Video S06: What is Immunotherapy? (Spanish). Video S07: What is Immunotherapy? (Punjabi). Video S08: What is Immunotherapy? (Cantonese). File S09: Your Guide to Cancer Immunotherapy.
